# THE CONCISE GUIDE TO PHARMACOLOGY 2021/22: Nuclear hormone receptors

**DOI:** 10.1111/bph.15540

**Published:** 2021-09-16

**Authors:** Stephen P H Alexander, John A Cidlowski, Eamonn Kelly, Alistair Mathie, John A Peters, Emma L Veale, Jane F Armstrong, Elena Faccenda, Simon D Harding, Adam J Pawson, Christopher Southan, Jamie A Davies, Laurel Coons, Peter J. Fuller, Kenneth S. Korach, Morag J. Young

**Affiliations:** ^1^ School of Life Sciences University of Nottingham Medical School Nottingham NG7 2UH, UK; ^2^ National Institute of Environmental Health Sciences, National Institutes of Health, Department of Health and Human Services Research Triangle Park NC 27709 USA; ^3^ School of Physiology, Pharmacology and Neuroscience University of Bristol Bristol BS8 1TD UK; ^4^ Medway School of Pharmacy, The Universities of Greenwich and Kent at Medway, Anson Building, Central Avenue Chatham Maritime Chatham, Kent ME4 4TB UK; ^5^ Neuroscience Division, Medical Education Institute, Ninewells Hospital and Medical School University of Dundee Dundee DD1 9SY UK; ^6^ Centre for Discovery Brain Sciences University of Edinburgh Edinburgh EH8 9XD UK; ^7^ Duke University Medical Center Durham, NC USA; ^8^ Hudson Institute of Medical Research Clayton Australia; ^9^ National Institute of Health Research Triangle Park NC USA; ^10^ Monash University Melbourne Australia

## Abstract

The Concise Guide to PHARMACOLOGY 2021/22 is the fifth in this series of biennial publications. The Concise Guide provides concise overviews, mostly in tabular format, of the key properties of nearly 1900 human drug targets with an emphasis on selective pharmacology (where available), plus links to the open access knowledgebase source of drug targets and their ligands (www.guidetopharmacology.org), which provides more detailed views of target and ligand properties. Although the Concise Guide constitutes over 500 pages, the material presented is substantially reduced compared to information and links presented on the website. It provides a permanent, citable, point‐in‐time record that will survive database updates. The full contents of this section can be found at http://onlinelibrary.wiley.com/doi/bph.15540. Nuclear hormone receptors are one of the six major pharmacological targets into which the Guide is divided, with the others being: G protein‐coupled receptors, catalytic receptors, enzymes and transporters. These are presented with nomenclature guidance and summary information on the best available pharmacological tools, alongside key references and suggestions for further reading. The landscape format of the Concise Guide is designed to facilitate comparison of related targets from material contemporary to mid‐2021, and supersedes data presented in the 2019/20, 2017/18, 2015/16 and 2013/14 Concise Guides and previous Guides to Receptors and Channels. It is produced in close conjunction with the Nomenclature and Standards Committee of the International Union of Basic and Clinical Pharmacology (NC‐IUPHAR), therefore, providing official IUPHAR classification and nomenclature for human drug targets, where appropriate.

## Conflict of interest

The authors state that there are no conflicts of interest to disclose.

## Overview

Nuclear receptors are specialised transcription factors with commonalities of sequence and structure, which bind as homo‐ or heterodimers to specific consensus sequences of DNA (response elements) in the promoter region of particular target genes. They regulate (either promoting or repressing) transcription of these target genes in response to a variety of endogenous ligands. Endogenous agonists are hydrophobic entities which, when bound to the receptor promote conformational changes in the receptor to allow recruitment (or dissociation) of protein partners, generating a large multiprotein complex.

Two major subclasses of nuclear receptors with identified endogenous agonists can be identified: steroid and non‐steroid hormone receptors. Steroid hormone receptors function typically as dimeric entities and are thought to be resident outside the nucleus in the unliganded state in a complex with chaperone proteins, which are liberated upon agonist binding. Migration to the nucleus and interaction with other regulators of gene transcription, including RNA polymerase, acetyltransferases and deacetylases, allows gene transcription to be regulated. Non‐steroid hormone receptors typically exhibit a greater distribution in the nucleus in the unliganded state and interact with other nuclear receptors to form heterodimers, as well as with other regulators of gene transcription, leading to changes in gene transcription upon agonist binding.

Selectivity of gene regulation is brought about through interaction of nuclear receptors with particular consensus sequences of DNA, which are arranged typically as repeats or inverted palindromes to allow accumulation of multiple transcription factors in the promoter regions of genes.

## Family structure

S247 1A. Thyroid hormone receptors

S248 1B. Retinoic acid receptors

S249 1C. Peroxisome proliferator‐activated receptors

S250 1D. Rev‐Erb receptors

S251 1F. Retinoic acid‐related orphans

S252 1H. Liver X receptor‐like receptors

S253 1I. Vitamin D receptor‐like receptors

S254 2A. Hepatocyte nuclear factor‐4 receptors

S255 2B. Retinoid X receptors

S255 2C. Testicular receptors

S256 2E. Tailless‐like receptors

S256 2F. COUP‐TF‐like receptors

S257 3B. Estrogen‐related receptors

S257 4A. Nerve growth factor IB‐like receptors

S258 5A. Fushi tarazu F1‐like receptors

S259 6A. Germ cell nuclear factor receptors

S259 0B. DAX‐like receptors

S260 Steroid hormone receptors

S260 3A. Estrogen receptors

S261 3C. 3‐Ketosteroid receptors

## 1A. Thyroid hormone receptors

### Overview

Thyroid hormone receptors (**TRs, nomenclature as agreed by the** **NC‐IUPHAR** **Subcommittee on Nuclear Hormone Receptors [** **1** **,** **40** **]**) are nuclear hormone receptors of the NR1A family, with diverse roles regulating macronutrient metabolism, cognition and cardiovascular homeostasis. TRs are activated by thyroxine (T_4_ ) and thyroid hormone (triiodothyronine). Once activated by a ligand, the receptor acts as a transcription factor either as a monomer, homodimer or heterodimer with members of the retinoid X receptor family. NH‐3 has been described as an antagonist at TRs with modest selectivity for TRβ [108].

### Further reading on 1A. Thyroid hormone receptors

Elbers LP *et al*. (2016) Thyroid Hormone Mimetics: the Past, Current Status and Future Challenges. *Curr Atheroscler Rep* **18**: 14 [PMID:26886134]

Flamant F *et al*. (2006) International Union of Pharmacology. LIX. The pharmacology and classification of the nuclear receptor superfamily: thyroid hormone receptors. *Pharmacol Rev* **58**: 705‐11 [PMID:17132849]

Mendoza A *et al*. (2017) New insights into thyroid hormone action. *Pharmacol Ther* **173**: 135‐145 [PMID:28174093]

**Table:** 

Nomenclature	Thyroid hormone receptor‐α	Thyroid hormone receptor‐β
Systematic nomenclature	NR1A1	NR1A2
HGNC, UniProt	*THRA* , P10827	*THRB* , P10828
Rank order of potency	triiodothyronine > T_4_	triiodothyronine > T_4_
Agonists	dextrothyroxine [19]	dextrothyroxine [19]
Selective agonists	–	sobetirome [24, 128]

### Comments

An interaction with integrin αVβ3 has been suggested to underlie plasma membrane localization of TRs and non‐genomic signalling [8].One splice variant, TRα_2_, lacks a functional DNA‐binding domain and appears to act as a transcription suppressor.

Although radioligand binding assays have been described for these receptors, the radioligands are not commercially available.

## 1B. Retinoic acid receptors

### Overview

Retinoic acid receptors (**nomenclature as agreed by the** **NC‐IUPHAR** **Subcommittee on Nuclear Hormone Receptors [** **1** **,** **46** **]**) are nuclear hormone receptors of the NR1B family activated by the vitamin A‐derived agonists tretinoin (ATRA) and alitretinoin, and the RAR‐selective synthetic agonists TTNPB and adapalene. BMS493 is a family‐selective antagonist [48].

### Further reading on 1B. Retinoic acid receptors

Duong V *et al*. (2011) The molecular physiology of nuclear retinoic acid receptors. From health to disease. *Biochim Biophys Acta* **1812**: 1023‐31 [PMID:20970498]

Germain P *et al*. (2006) International Union of Pharmacology. LX. Retinoic acid receptors. *Pharmacol Rev* **58**: 712‐25 [PMID:17132850]

Larange A *et al*. (2016) Retinoic Acid and Retinoic Acid Receptors as Pleiotropic Modulators of the Immune System. *Annu Rev Immunol* **34**: 369‐94 [PMID:27168242]

Saeed A *et al*. (2017) The interrelationship between bile acid and vitamin A homeostasis. *Biochim Biophys Acta* **1862**: 496‐512 [PMID:28111285]

**Table:** 

Nomenclature	Retinoic acid receptor‐α	Retinoic acid receptor‐β	Retinoic acid receptor‐γ
Systematic nomenclature	NR1B1	NR1B2	NR1B3
HGNC, UniProt	*RARA* , P10276	*RARB* , P10826	*RARG* , P13631
Agonists	tretinoin [23]	tretinoin [23]	tretinoin [23]
Sub/family‐selective agonists	tazarotene [23]	tazarotene [23], adapalene [22]	tazarotene [23], adapalene [22]
Selective agonists	BMS753 [45], tamibarotene [146], Ro 40‐6055 [31]	AC261066 [87], AC55649 [86, 87]	AHPN [22]
Selective antagonists	Ro 41‐5253 (pIC_50_ 6.3–7.2) [2, 68]	–	MM 11253 [75]

### Comments

Ro 41‐5253 has been suggested to be a PPARγ agonist [127]. LE135 is an antagonist with selectivity for RARα and RARβ compared with RARγ [83].

## 1C. Peroxisome proliferator‐activated receptors

### Overview

Peroxisome proliferator‐activated receptors (**PPARs, nomenclature as agreed by the** **NC‐IUPHAR** **Subcommittee on Nuclear Hormone Receptors [** **1** **,** **99** **]**) are nuclear hormone receptors of the NR1C family, with diverse roles regulating lipid homeostasis, cellular differentiation, proliferation and the immune response. PPARs have many potential endogenous agonists [13, 99], including 15‐deoxy‐Δ^12,14^‐PGJ_2_ , prostacyclin (PGI_2_ ), many fatty acids and their oxidation products, lysophosphatidic acid (LPA) [96], 13‐HODE, 15S‐HETE, Paz‐PC, azelaoyl‐PAF and leukotriene B4 (LTB_4_ ). Bezafibrate acts as a non‐selective agonist for the PPAR family [155]. These receptors also bind hypolipidaemic drugs (PPARα) and anti‐diabetic thiazolidinediones (PPARγ), as well as many non‐steroidal anti‐inflammatory drugs, such as sulindac and indomethacin. Once activated by a ligand, the receptor forms a heterodimer with members of the retinoid X receptor family and can act as a transcription factor. Although radioligand binding assays have been described for all three receptors, the radioligands are not commercially available. Commonly, receptor occupancy studies are conducted using fluorescent ligands and truncated forms of the receptor limited to the ligand binding domain.

### Further reading on 1C. Peroxisome proliferator‐activated receptors

Cheang WS *et al*. (2015) The peroxisome proliferator‐activated receptors in cardiovascular diseases: experimental benefits and clinical challenges. *Br J Pharmacol* **172**: 5512‐22 [PMID:25438608]

Gross B *et al*. (2017) PPARs in obesity‐induced T2DM, dyslipidaemia and NAFLD. *Nat Rev Endocrinol* **13**: 36‐49 [PMID:27636730]

Hallenborg P *et al*. (2016) The elusive endogenous adipogenic PPARγ agonists: Lining up the suspects. *Prog Lipid Res* **61**: 149‐62 [PMID:26703188]

Michalik L *et al*. (2006) International Union of Pharmacology. LXI. Peroxisome proliferator‐activated receptors. *Pharmacol Rev* **58**: 726‐41 [PMID:17132851]

Sauer S. (2015) Ligands for the Nuclear Peroxisome Proliferator‐Activated Receptor Gamma. *Trends Pharmacol Sci* **36**: 688‐704 [PMID:26435213]

**Table:** 

Nomenclature	Peroxisome proliferator‐activated receptor‐α	Peroxisome proliferator‐activated receptor‐β/δ	Peroxisome proliferator‐activated receptor‐γ
Systematic nomenclature	NR1C1	NR1C2	NR1C3
HGNC, UniProt	*PPARA* , Q07869	*PPARD* , Q03181	*PPARG* , P37231
Selective agonists	GW7647 [17, 18], CP‐775146 [66], pirinixic acid [155], gemfibrozil [29]	GW0742X [51, 140], GW501516 [110]	GW1929 [17], bardoxolone (Partial agonist) [149], rosiglitazone [58, 79, 161], troglitazone [58, 161], pioglitazone [58, 125, 161], ciglitazone [58]
Selective antagonists	GW6471 (pIC_50_ 6.6) [158]	GSK0660 (pIC_50_ 6.5) [129]	T0070907 (pK_i_ 9) [76], GW9662 (Irreversible inhibition) (pIC_50_ 8.1) [77], CDDO‐Me (pK_i_ 6.9) [149]

### Comments

As with the estrogen receptor antagonists, many agents show tissue‐selective efficacy (*e.g*. [12, 107, 122]). Agonists with mixed activity at PPARα and PPARγ have also been described (*e.g* [32, 54, 159]).

## 1D. Rev‐Erb receptors

### Overview

Rev‐erb receptors (**nomenclature as agreed by the** **NC‐IUPHAR** **Subcommittee on Nuclear Hormone Receptors [** **1** **,** **7** **]**) have yet to be officially paired with an endogenous ligand, but are thought to be activated by heme.

### Further reading on 1D. Rev‐Erb receptors

Benoit G *et al*. (2006) International Union of Pharmacology. LXVI. Orphan nuclear receptors. *Pharmacol Rev* **58**: 798‐836 [PMID:17132856]

Gonzalez‐Sanchez E *et al*. (2015) Nuclear receptors in acute and chronic cholestasis. *Dig Dis* **33**: 357‐66 [PMID:26045270]

Gustafson CL *et al*. (2015) Emerging models for the molecular basis of mammalian circadian timing. *Biochemistry* **54**: 134‐49 [PMID:25303119]

Sousa EH *et al*. (2017) Drug discovery targeting heme‐based sensors and their coupled activities. *J Inorg Biochem* **167**: 12‐20 [PMID:27893989]

**Table:** 

Nomenclature	Rev‐Erb‐α	Rev‐Erb‐β
Systematic nomenclature	NR1D1	NR1D2
HGNC, UniProt	*NR1D1* , P20393	*NR1D2* , Q14995
Endogenous agonists	heme [119, 160]	heme [95, 119, 160]
Selective agonists	GSK4112 [52], GSK4112 [71]	–
Selective antagonists	SR8278 (pIC_50_ 6.5) [71]	–

## 1F. Retinoic acid‐related orphans

### Overview

Retinoic acid receptor‐related orphan receptors (ROR, **nomenclature as agreed by the** **NC‐IUPHAR** **Subcommittee on Nuclear Hormone Receptors [** **1** **,** **7** **]**) have yet to be assigned a definitive endogenous ligand, although RORα may be synthesized with a ‘captured’ agonist such as cholesterol [64, 65].

### Further reading on 1F. Retinoic acid‐related orphans

Benoit G *et al*. (2006) International Union of Pharmacology. LXVI. Orphan nuclear receptors. *Pharmacol Rev* **58**: 798‐836 [PMID:17132856]

Cyr P *et al*. (2016) Recent progress on nuclear receptor RORγ modulators. *Bioorg Med Chem Lett* **26**: 4387‐4393 [PMID:27542308]

Germain P *et al*. (2006) Overview of nomenclature of nuclear receptors. *Pharmacol Rev* **58**: 685‐704 [PMID:17132848]

Guillemot‐Legris O *et al*. (2016) Oxysterols in Metabolic Syndrome: From Bystander Molecules to Bioactive Lipids. *Trends Mol Med* **22**: 594‐614 [PMID:27286741]

Mutemberezi V *et al*. (2016) Oxysterols: From cholesterol metabolites to key mediators. *Prog Lipid Res* **64**: 152‐169 [PMID:27687912]

**Table:** 

Nomenclature	RAR‐related orphan receptor‐α	RAR‐related orphan receptor‐β	RAR‐related orphan receptor‐γ
Systematic nomenclature	NR1F1	NR1F2	NR1F3
HGNC, UniProt	*RORA* , P35398	*RORB* , Q92753	*RORC* , P51449
Endogenous agonists	cholesterol [65, 112]	–	–
Selective agonists	7‐hydroxycholesterol [14], cholesterol sulphate [14, 65]	–	–
Comments	–	–	The immune system function of RORC proteins most likely resides with expression of the RORγt isoform by immature CD4^+^/CD8^+^ cells in the thymus [34, 139] and in lymphoid tissue inducer (LTi) cells [35].

### Comments

Tretinoin shows selectivity for RORβ within the ROR family [134]. RORα has been suggested to be a nuclear receptor responding to melatonin[154].

## 1H. Liver X receptor‐like receptors

### Overview

Liver X and farnesoid X receptors (LXR and FXR, **nomenclature as agreed by the** **NC‐IUPHAR** **Subcommittee on Nuclear Hormone Receptors [** **1** **,** **103** **]**) are members of a steroid analogue‐activated nuclear receptor subfamily, which form heterodimers with members of the retinoid X receptor family. Endogenous ligands for LXRs include hydroxycholesterols (OHC), while FXRs appear to be activated by bile acids. In humans and primates, *NR1H5P* is a pseudogene. However, in other mammals, it encodes a functional nuclear hormone receptor that appears to be involved in cholesterol biosynthesis [111].

### Further reading on 1H. Liver X receptor‐like receptors

Courtney R *et al*. (2016) LXR Regulation of Brain Cholesterol: From Development to Disease. *Trends Endocrinol Metab* **27**: 404‐414 [PMID:27113081]

El‐Gendy BEM *et al*. (2018) Recent Advances in the Medicinal Chemistry of Liver X Receptors. *J Med Chem* **61**: 10935‐10956 [PMID:30004226]

Gadaleta RM *et al*. (2010) Bile acids and their nuclear receptor FXR: Relevance for hepatobiliary and gastrointestinal disease. *Biochim Biophys Acta* **1801**: 683‐92 [PMID:20399894]

Merlen G *et al*. (2017) Bile acids and their receptors during liver regeneration: "Dangerous protectors". *Mol Aspects Med* **56**: 25‐33 [PMID:28302491]

Moore DD *et al*. (2006) International Union of Pharmacology. LXII. The NR1H and NR1I receptors: constitutive androstane receptor, pregnene X receptor, farnesoid X receptor alpha, farnesoid X receptor beta, liver X receptor alpha, liver X receptor beta, and vitamin D receptor. *Pharmacol Rev* **58**: 742‐59 [PMID:17132852]

Mouzat K *et al*. (2016) Liver X receptors: from cholesterol regulation to neuroprotection‐a new barrier against neurodegeneration in amyotrophic lateral sclerosis? *Cell Mol Life Sci* **73**: 3801‐8 [PMID:27510420]

Schulman IG. (2017) Liver X receptors link lipid metabolism and inflammation. *FEBS Lett* **591**: 2978‐2991 [PMID:28555747]

**Table:** 

Nomenclature	Farnesoid X receptor	Farnesoid X receptor‐β	Liver X receptor‐α	Liver X receptor‐β
Systematic nomenclature	NR1H4	NR1H5	NR1H3	NR1H2
HGNC, UniProt	*NR1H4* , Q96RI1	*NR1H5P* , –	*NR1H3* , Q13133	*NR1H2* , P55055
Potency order	chenodeoxycholic acid > lithocholic acid, deoxycholic acid [90, 113]	–	20S‐hydroxycholesterol, 22R‐hydroxycholesterol, 24(S)‐hydroxycholesterol > 25‐hydroxycholesterol, 27‐hydroxycholesterol [78]	20S‐hydroxycholesterol, 22R‐hydroxycholesterol, 24(S)‐hydroxycholesterol > 25‐hydroxycholesterol, 27‐hydroxycholesterol [78]
Endogenous agonists	–	lanosterol [111] – Mouse	–	–
Selective agonists	GW4064 [92], obeticholic acid [114], fexaramine [33]	–	–	–
Selective antagonists	guggulsterone (pIC_50_ 5.7–6) [157]	–	–	–

### Comments

T0901317[120] and GW3965[25] are synthetic agonists acting at both LXRα and LXRβ with less than 10‐fold selectivity.

## 1I. Vitamin D receptor‐like receptors

### Overview

Vitamin D (VDR), Pregnane X (PXR) and Constitutive Androstane (CAR) receptors (**nomenclature as agreed by the** **NC‐IUPHAR** **Subcommittee on Nuclear Hormone Receptors [** **1** **,** **103** **]**) are members of the NR1I family of nuclear receptors, which form heterodimers with members of the retinoid X receptor family. PXR and CAR are activated by a range of exogenous compounds, with no established endogenous physiological agonists, although high concentrations of bile acids and bile pigments activate PXR and CAR [103].

### Further reading on 1I. Vitamin D receptor‐like receptors

Benoit G *et al*. (2006) International Union of Pharmacology. LXVI. Orphan nuclear receptors. *Pharmacol Rev* **58**: 798‐836 [PMID:17132856]

Long MD *et al*. (2015) Vitamin D receptor and RXR in the post‐genomic era. *J Cell Physiol* **230**: 758‐66 [PMID:25335912]

Moore DD *et al*. (2006) International Union of Pharmacology. LXII. The NR1H and NR1I receptors: constitutive androstane receptor, pregnene X receptor, farnesoid X receptor alpha, farnesoid X receptor beta, liver X receptor alpha, liver X receptor beta, and vitamin D receptor. *Pharmacol Rev* **58**: 742‐59 [PMID:17132852]

**Table:** 

Nomenclature	Vitamin D receptor	Pregnane X receptor	Constitutive androstane receptor
Systematic nomenclature	NR1I1	NR1I2	NR1I3
HGNC, UniProt	*VDR* , P11473	*NR1I2* , O75469	*NR1I3* , Q14994
Endogenous agonists	1,25‐dihydroxyvitamin D3 [11, 38]	17β‐estradiol [63]	–
Selective agonists	seocalcitol [26, 153], doxercalciferol	hyperforin [104, 152], 5β‐pregnane‐3,20‐dione [63], lovastatin [80], rifampicin [15, 80]	TCPOBOP [144] – Mouse, CITCO [89]
Selective antagonists	TEI‐9647 (pIC_50_ 8.2) [124] – Chicken, ZK159222 (pIC_50_ 7.5) [41, 59]	–	–
Comments	–	–	Clotrimazole [105] and T0901317 [67] although acting at other sites, function as antagonists of the constitutive androstane receptor.

## 2A. Hepatocyte nuclear factor‐4 receptors

### Overview

The nomenclature of hepatocyte nuclear factor‐4 receptors is agreed by the **NC‐IUPHAR** **Subcommittee on Nuclear Hormone Receptors [** **1** **,** **7** **]**. While linoleic acid has been identified as the endogenous ligand for HNF4α its function remains ambiguous [163]. HNF4γ has yet to be paired with an endogenous ligand.

### Further reading on 2A. Hepatocyte nuclear factor‐4 receptors

Benoit G *et al*. (2006) International Union of Pharmacology. LXVI. Orphan nuclear receptors. *Pharmacol Rev* **58**: 798‐836 [PMID:17132856]

Garattini E *et al*. (2016) Lipid‐sensors, enigmatic‐orphan and orphan nuclear receptors as therapeutic targets in breast‐cancer. *Oncotarget* **7**: 42661‐42682 [PMID:26894976]

Germain P *et al*. (2006) Overview of nomenclature of nuclear receptors. *Pharmacol Rev* **58**: 685‐704 [PMID:17132848]

Lu H. (2016) Crosstalk of HNF4*α* with extracellular and intracellular signaling pathways in the regulation of hepatic metabolism of drugs and lipids. *Acta Pharm Sin B* **6**: 393‐408 [PMID:27709008]

Walesky C *et al*. (2015) Role of hepatocyte nuclear factor 4α (HNF4α) in cell proliferation and cancer. *Gene Expr* **16**: 101‐8 [PMID:25700366]

**Table:** 

Nomenclature	Hepatocyte nuclear factor‐4‐α	Hepatocyte nuclear factor‐4‐γ
Systematic nomenclature	NR2A1	NR2A2
HGNC, UniProt	*HNF4A* , P41235	*HNF4G* , Q14541
Endogenous agonists	linoleic acid [163]	–
Selective antagonists	BI6015 [70]	–
Comments	HNF4α has constitutive transactivation activity [163] and binds DNA as a homodimer [62].	–

## 2B. Retinoid X receptors

### Overview

Retinoid X receptors (**nomenclature as agreed by the** **NC‐IUPHAR** **Subcommittee on Nuclear Hormone Receptors [** **1** **,** **47** **]**) are NR2B family members activated by alitretinoin and the RXR‐selective agonists bexarotene and LG100268, sometimes referred to as rexinoids. UVI3003[106] and HX531 [36] have been described as a pan‐RXR antagonists. These receptors form RXR‐RAR heterodimers and RXR‐RXR homodimers [21, 94].

### Further reading on 2B. Retinoid X receptors

Germain P *et al*. (2006) International Union of Pharmacology. LXIII. Retinoid X receptors. *Pharmacol Rev* **58**: 760‐72 [PMID:17132853]

Long MD *et al*. (2015) Vitamin D receptor and RXR in the post‐genomic era. *J Cell Physiol* **230**: 758‐66 [PMID:25335912]

Menéndez‐Gutiérrez MP *et al*. (2017) The multi‐faceted role of retinoid X receptor in bone remodeling. *Cell Mol Life Sci* **74**: 2135‐2149 [PMID:28105491]

**Table:** 

Nomenclature	Retinoid X receptor‐α	Retinoid X receptor‐β	Retinoid X receptor‐γ
Systematic nomenclature	NR2B1	NR2B2	NR2B3
HGNC, UniProt	*RXRA* , P19793	*RXRB* , P28702	*RXRG* , P48443
Sub/family‐selective agonists	bexarotene [16, 20, 141]	bexarotene [16, 20, 141]	bexarotene [16, 20, 141]
Selective agonists	CD3254 [49]	–	–

## 2C. Testicular receptors

### Overview

Testicular receptors (**nomenclature as agreed by the** **NC‐IUPHAR** **Subcommittee on Nuclear Hormone Receptors [** **7** **]**) have yet to be officially paired with an endogenous ligand, although testicular receptor 4 has been reported to respond to retinoids.

### Further reading on 2C. Testicular receptors

Benoit G *et al*. (2006) International Union of Pharmacology. LXVI. Orphan nuclear receptors. *Pharmacol Rev* **58**: 798‐836 [PMID:17132856]

Germain P *et al*. (2006) Overview of nomenclature of nuclear receptors. *Pharmacol Rev* **58**: 685‐704 [PMID:17132848]

Safe S *et al*. (2014) Minireview: role of orphan nuclear receptors in cancer and potential as drug targets. *Mol Endocrinol* **28**: 157‐72 [PMID:24295738]

Wu D *et al*. (2016) The emerging roles of orphan nuclear receptors in prostate cancer. *Biochim Biophys Acta* **1866**: 23‐36 [PMID:27264242]

**Table:** 

Nomenclature	Testicular receptor 2	Testicular receptor 4
Systematic nomenclature	NR2C1	NR2C2
HGNC, UniProt	*NR2C1* , P13056	*NR2C2* , P49116
Endogenous agonists	–	retinol [169], tretinoin [169]
Comments	Forms a heterodimer with TR4; gene disruption appears without effect on testicular development or function [130].	Forms a heterodimer with TR2.

## 2E. Tailless‐like receptors

### Overview

Tailless‐like receptors (**nomenclature as agreed by the** **NC‐IUPHAR** **Subcommittee on Nuclear Hormone Receptors [** **7** **]**) have yet to be officially paired with an endogenous ligand.

### Further reading on 2E. Tailless‐like receptors

Benod C *et al*. (2016) TLX: An elusive receptor. *J Steroid Biochem Mol Biol* **157**: 41‐7 [PMID:26554934]

Benoit G *et al*. (2006) International Union of Pharmacology. LXVI. Orphan nuclear receptors. *Pharmacol Rev* **58**: 798‐836 [PMID:17132856]

Germain P *et al*. (2006) Overview of nomenclature of nuclear receptors. *Pharmacol Rev* **58**: 685‐704 [PMID:17132848]

O'Leary JD *et al*. (2018) Regulation of behaviour by the nuclear receptor TLX. *Genes Brain Behav* **17**: e12357 [PMID:27790850]

**Table:** 

Nomenclature	TLX	PNR
Systematic nomenclature	NR2E1	NR2E3
HGNC, UniProt	*NR2E1* , Q9Y466	*NR2E3* , Q9Y5X4
Agonists	BMS493 [53], tretinoin [53]	–
Comments	Gene disruption is associated with abnormal brain development [74, 102].	–

## 2F. COUP‐TF‐like receptors

### Overview

COUP‐TF‐like receptors (**nomenclature as agreed by the** **NC‐IUPHAR** **Subcommittee on Nuclear Hormone Receptors [** **1** **,** **7** **]**) have yet to be officially paired with an endogenous ligand.

### Further reading on 2F. COUP‐TF‐like receptors

Benoit G *et al*. (2006) International Union of Pharmacology. LXVI. Orphan nuclear receptors. *Pharmacol Rev* **58**: 798‐836 [PMID:17132856]

Germain P *et al*. (2006) Overview of nomenclature of nuclear receptors. *Pharmacol Rev* **58**: 685‐704 [PMID:17132848]

Wu D *et al*. (2016) The emerging roles of orphan nuclear receptors in prostate cancer. *Biochim Biophys Acta* **1866**: 23‐36 [PMID:27264242]

Wu SP *et al*. (2016) Choose your destiny: Make a cell fate decision with COUP‐TFII. *J Steroid Biochem Mol Biol* **157**: 7‐12 [PMID:26658017]

**Table:** 

Nomenclature	COUP‐TF1	COUP‐TF2	V‐erbA‐related gene
Systematic nomenclature	NR2F1	NR2F2	NR2F6
HGNC, UniProt	*NR2F1* , P10589	*NR2F2* , P24468	*NR2F6* , P10588
Comments	Gene disruption is perinatally lethal [118].	Gene disruption is embryonically lethal [115].	Gene disruption impairs CNS development [151].

## 3B. Estrogen‐related receptors

### Overview

Estrogen‐related receptors (**nomenclature as agreed by the** **NC‐IUPHAR** **Subcommittee on Nuclear Hormone Receptors [** **7** **]**) have yet to be officially paired with an endogenous ligand.

### Further reading on 3B. Estrogen‐related receptors

Benoit G *et al*. (2006) International Union of Pharmacology. LXVI. Orphan nuclear receptors. *Pharmacol Rev* **58**: 798‐836 [PMID:17132856]

Divekar SD *et al*. (2016) Estrogen‐related receptor β (ERRβ) ‐ renaissance receptor or receptor renaissance? *Nucl Recept Signal* **14**: e002 [PMID:27507929]

Germain P *et al*. (2006) Overview of nomenclature of nuclear receptors. *Pharmacol Rev* **58**: 685‐704 [PMID:17132848]

Tam IS *et al*. (2016) There and back again: The journey of the estrogen‐related receptors in the cancer realm. *J Steroid Biochem Mol Biol* **157**: 13‐9 [PMID:26151739]

Wu D *et al*. (2016) The emerging roles of orphan nuclear receptors in prostate cancer. *Biochim Biophys Acta* **1866**: 23‐36 [PMID:27264242]

**Table:** 

Nomenclature	Estrogen‐related receptor‐α	Estrogen‐related receptor‐β	Estrogen‐related receptor‐γ
Systematic nomenclature	NR3B1	NR3B2	NR3B3
HGNC, UniProt	*ESRRA* , P11474	*ESRRB* , O95718	*ESRRG* , P62508
Comments	Activated by some dietary flavonoids [136]; activated by the synthetic agonist GSK4716 [172] and blocked by XCT790 [156].	May be activated by DY131 [162].	May be activated by DY131 [162].

## 4A. Nerve growth factor IB‐like receptors

### Overview

Nerve growth factor IB‐like receptors (**nomenclature as agreed by the** **NC‐IUPHAR** **Subcommittee on Nuclear Hormone Receptors [** **7** **]**) have yet to be officially paired with an endogenous ligand.

### Further reading on 4A. Nerve growth factor IB‐like receptors

Benoit G *et al*. (2006) International Union of Pharmacology. LXVI. Orphan nuclear receptors. *Pharmacol Rev* **58**: 798‐836 [PMID:17132856]

Germain P *et al*. (2006) Overview of nomenclature of nuclear receptors. *Pharmacol Rev* **58**: 685‐704 [PMID:17132848]

Ranhotra HS. (2015) The NR4A orphan nuclear receptors: mediators in metabolism and diseases. *J Recept Signal Transduct Res* **35**: 184‐8 [PMID:25089663]

Rodríguez‐Calvo R *et al*. (2017) The NR4A subfamily of nuclear receptors: potential new therapeutic targets for the treatment of inflammatory diseases. *Expert Opin Ther Targets* **21**: 291‐304 [PMID:28055275]

Safe S *et al*. (2016) Nuclear receptor 4A (NR4A) family ‐ orphans no more. *J Steroid Biochem Mol Biol* **157**: 48‐60 [PMID:25917081]

**Table:** 

Nomenclature	Nerve Growth factor IB	Nuclear receptor related 1	Neuron‐derived orphan receptor 1
Systematic nomenclature	NR4A1	NR4A2	NR4A3
HGNC, UniProt	*NR4A1* , P22736	*NR4A2* , P43354	*NR4A3* , Q92570
Comments	An endogenous agonist, cytosporone B, has been described [164], although structural analysis and molecular modelling has not identified a ligand binding site [4, 39, 150].	–	–

## 5A. Fushi tarazu F1‐like receptors

### Overview

Fushi tarazu F1‐like receptors (**nomenclature as agreed by the** **NC‐IUPHAR** **Subcommittee on Nuclear Hormone Receptors [** **7** **]**) have yet to be officially paired with an endogenous ligand.

### Further reading on 5A. Fushi tarazu F1‐like receptors

Benoit G *et al*. (2006) International Union of Pharmacology. LXVI. Orphan nuclear receptors. *Pharmacol Rev* **58**: 798‐836 [PMID:17132856]

Garattini E *et al*. (2016) Lipid‐sensors, enigmatic‐orphan and orphan nuclear receptors as therapeutic targets in breast‐cancer. *Oncotarget* **7**: 42661‐42682 [PMID:26894976]

Germain P *et al*. (2006) Overview of nomenclature of nuclear receptors. *Pharmacol Rev* **58**: 685‐704 [PMID:17132848]

Zhi X *et al*. (2016) Structures and regulation of non‐X orphan nuclear receptors: A retinoid hypothesis. *J Steroid Biochem Mol Biol* **157**: 27‐40 [PMID:26159912]

Zimmer V *et al*. (2015) Nuclear receptor variants in liver disease. *Dig Dis* **33**: 415‐9 [PMID:26045277]

**Table:** 

Nomenclature	Steroidogenic factor 1	Liver receptor homolog‐1
Systematic nomenclature	NR5A1	NR5A2
HGNC, UniProt	*NR5A1* , Q13285	*NR5A2* , O00482
Comments	Reported to be inhibited by AC45594 [30] and SID7969543 [88].	–

## 6A. Germ cell nuclear factor receptors

### Overview

Germ cell nuclear factor receptors (**nomenclature as agreed by the** **NC‐IUPHAR** **Subcommittee on Nuclear Hormone Receptors [** **7** **]**) have yet to be officially paired with an endogenous ligand.

### Further reading on 6A. Germ cell nuclear factor receptors

Benoit G *et al*. (2006) International Union of Pharmacology. LXVI. Orphan nuclear receptors. *Pharmacol Rev* **58**: 798‐836 [PMID:17132856]

Garattini E *et al*. (2016) Lipid‐sensors, enigmatic‐orphan and orphan nuclear receptors as therapeutic targets in breast‐cancer. *Oncotarget* **7**: 42661‐42682 [PMID:26894976]

Germain P *et al*. (2006) Overview of nomenclature of nuclear receptors. *Pharmacol Rev* **58**: 685‐704 [PMID:17132848]

Safe S *et al*. (2014) Minireview: role of orphan nuclear receptors in cancer and potential as drug targets. *Mol Endocrinol* **28**: 157‐72 [PMID:24295738]

Zhi X *et al*. (2016) Structures and regulation of non‐X orphan nuclear receptors: A retinoid hypothesis. *J Steroid Biochem Mol Biol* **157**: 27‐40 [PMID:26159912]

**Table:** 

Nomenclature	Germ cell nuclear factor
Systematic nomenclature	NR6A1
HGNC, UniProt	*NR6A1* , Q15406

## 0B. DAX‐like receptors

### Overview

Dax‐like receptors (**nomenclature as agreed by the** **NC‐IUPHAR** **Subcommittee on Nuclear Hormone Receptors [** **7** **]**) have yet to be officially paired with an endogenous ligand.

### Further reading on 0B. DAX‐like receptors

Benoit G *et al*. (2006) International Union of Pharmacology. LXVI. Orphan nuclear receptors. *Pharmacol Rev* **58**: 798‐836 [PMID:17132856]

Garattini E *et al*. (2016) Lipid‐sensors, enigmatic‐orphan and orphan nuclear receptors as therapeutic targets in breast‐cancer. *Oncotarget* **7**: 42661‐42682 [PMID:26894976]

Germain P *et al*. (2006) Overview of nomenclature of nuclear receptors. *Pharmacol Rev* **58**: 685‐704 [PMID:17132848]

Safe S *et al*. (2014) Minireview: role of orphan nuclear receptors in cancer and potential as drug targets. *Mol Endocrinol* **28**: 157‐72 [PMID:24295738]

Wu D *et al*. (2016) The emerging roles of orphan nuclear receptors in prostate cancer. *Biochim Biophys Acta* **1866**: 23‐36 [PMID:27264242]

**Table:** 

Nomenclature	DAX1	SHP
Systematic nomenclature	NR0B1	NR0B2
HGNC, UniProt	*NR0B1* , P51843	*NR0B2* , Q15466

## Steroid hormone receptors

### Overview

Steroid hormone receptors (**nomenclature as agreed by the** **NC‐IUPHAR** **Subcommittee on Nuclear Hormone Receptors [** **1** **,** **28** **,** **85** **]**) are nuclear hormone receptors of the NR3 class, with endogenous agonists that may be divided into 3‐hydroxysteroids (estrone and 17β‐estradiol) and 3‐ketosteroids (dihydrotestosterone[DHT], aldosterone, cortisol, corticosterone, progesterone and testosterone). These receptors exist as dimers coupled with chaperone molecules (such as hsp90β( *HSP90AB1* , P08238) and immunophilin FKBP52: *FKBP4* , Q02790), which are shed on binding the steroid hormone. Although rapid signalling phenomena are observed [82, 117], the principal signalling cascade appears to involve binding of the activated receptors to nuclear hormone response elements of the genome, with a 15‐nucleotide consensus sequence AGAACAnnnTGTTCT (*i.e.* an inverted palindrome) as homo‐ or heterodimers. They also affect transcription by protein‐protein interactions with other transcription factors, such as activator protein 1 (AP‐1) and nuclear factor κB (NF‐κB). Splice variants of each of these receptors can form functional or non‐functional monomers that can dimerize to form functional or non‐functional receptors. For example, alternative splicing of PR mRNA produces A and B monomers that combine to produce functional AA, AB and BB receptors with distinct characteristics [145].

A 7TM receptor responsive to estrogen ( *GPER1* , Q99527, also known as GPR30, see [116]) has been described. Human orthologues of 7TM 'membrane progestin receptors' ( *PAQR7* , *PAQR8* and *PAQR5* ), initially discovered in fish [170, 171], appear to localize to intracellular membranes and respond to 'non‐genomic' progesterone analogues independently of G proteins [132].

## 3A. Estrogen receptors

### Overview

Estrogen receptor (ER) activity regulates diverse physiological processes *via* transcriptional modulation of target genes [1]. The selection of target genes and the magnitude of the response, be it induction or repression, are determined by many factors, including the effect of the hormone ligand and DNA binding on ER structural conformation, and the local cellular regulatory environment. The cellular environment defines the specific complement of DNA enhancer and promoter elements present and the availability of coregulators to form functional transcription complexes. Together, these determinants control the resulting biological response.

### Further reading on 3A. Estrogen receptors

Coons LA *et al*. (2017) DNA Sequence Constraints Define Functionally Active Steroid Nuclear Receptor Binding Sites in Chromatin. *Endocrinology* **158**: 3212‐3234 [PMID:28977594]

Dahlman‐Wright K *et al*. (2006) International Union of Pharmacology. LXIV. Estrogen receptors. *Pharmacol Rev* **58**: 773‐81 [PMID:17132854]

Gonzalez‐Sanchez E *et al*. (2015) Nuclear receptors in acute and chronic cholestasis. *Dig Dis* **33**: 357‐66 [PMID:26045270]

Hewitt SC *et al*. (2016) What's new in estrogen receptor action in the female reproductive tract. *J Mol Endocrinol* **56**: R55‐71 [PMID:26826253]

Jameera Begam A *et al*. (2017) Estrogen receptor agonists/antagonists in breast cancer therapy: A critical review. *Bioorg Chem* **71**: 257‐274 [PMID:28274582]

Warner M *et al*. (2017) Estrogen Receptor β as a Pharmaceutical Target. *Trends Pharmacol Sci* **38**: 92‐99 [PMID:27979317]

**Table:** 

Nomenclature	Estrogen receptor‐α	Estrogen receptor‐β
Systematic nomenclature	NR3A1	NR3A2
HGNC, UniProt	*ESR1* , P03372	*ESR2* , Q92731
Endogenous agonists	estriol [73], estrone [73]	–
Selective agonists	propylpyrazoletriol [72, 133], ethinylestradiol [61]	WAY200070 [91], diarylpropionitrile [98, 133], prinaberel [27, 91]
Sub/family‐selective antagonists	bazedoxifene (pIC_50_ 7.6) [101]	bazedoxifene (pIC_50_ 7.1) [101]
Selective antagonists	clomiphene (pK_i_ 8.9) [3], methyl‐piperidino‐pyrazole (pK_i_ 8.6) [137]	R,R‐THC (pK_i_ 8.4) [97, 138], PHTPP (pK_i_ 6.9) [168]

### Comments

R,R‐THC exhibits partial agonist activity at ERα [97, 138]. Estrogen receptors may be blocked non‐selectively by tamoxifen and raloxifene and labelled by [^3^H]17β‐estradiol and [^3^H]tamoxifen. Many agents thought initially to be antagonists at estrogen receptors appear to have tissue‐specific efficacy (*e.g*. Tamoxifen is an antagonist at estrogen receptors in the breast, but is an agonist at estrogen receptors in the uterus), hence the descriptor SERM (selective estrogen receptor modulator) or SnuRM (selective nuclear receptor modulator). Y134 has been suggested to be an ERα‐selective estrogen receptor modulator [109].

## 3C. 3‐Ketosteroid receptors

### Overview

Steroid hormone receptors (**nomenclature as agreed by the** **NC‐IUPHAR** **Subcommittee on Nuclear Hormone Receptors [** **1** **,** **28** **,** **85** **]**) are nuclear hormone receptors of the NR3 class, with endogenous agonists that may be divided into 3‐hydroxysteroids (estrone and 17β‐estradiol) and 3‐ketosteroids (dihydrotestosterone[DHT], aldosterone, cortisol, corticosterone, progesterone and testosterone). For rodent GR and MR, the physiological ligand is corticosterone rather than cortisol.

### Further reading on 3C. 3‐Ketosteroid receptors

Baker ME *et al*. (2017) 30 YEARS OF THE MINERALOCORTICOID RECEPTOR: Evolution of the mineralocorticoid receptor: sequence, structure and function. *J Endocrinol* **234**: T1‐T16 [PMID:28468932]

Carroll JS *et al*. (2017) Deciphering the divergent roles of progestogens in breast cancer. *Nat Rev Cancer* **17**: 54‐64 [PMID:27885264]

Cohen DM *et al*. (2017) Nuclear Receptor Function through Genomics: Lessons from the Glucocorticoid Receptor. *Trends Endocrinol Metab* **28**: 531‐540 [PMID:28495406]

de Kloet ER *et al*. (2017) Brain mineralocorticoid receptor function in control of salt balance and stress‐adaptation. *Physiol Behav* **178**: 13‐20 [PMID:28089704]

Garg D *et al*. (2017) Progesterone‐Mediated Non‐Classical Signaling. *Trends Endocrinol Metab* **28**: 656‐668 [PMID:28651856]

Lu NZ *et al*. (2006) International Union of Pharmacology. LXV. The pharmacology and classification of the nuclear receptor superfamily: glucocorticoid, mineralocorticoid, progesterone, and androgen receptors. *Pharmacol Rev* **58**: 782‐97 [PMID:17132855]

Lucas‐Herald AK *et al*. (2017) Genomic and non‐genomic effects of androgens in the cardiovascular system: clinical implications. *Clin Sci* **131**: 1405‐1418 [PMID:28645930]

Wadosky KM *et al*. (2017) Androgen receptor splice variants and prostate cancer: From bench to bedside. *Oncotarget* **8**: 18550‐18576 [PMID:28077788]

Weikum ER *et al*. (2017) Glucocorticoid receptor control of transcription: precision and plasticity via allostery. *Nat Rev Mol Cell Biol* **18**: 159‐174 [PMID:28053348]

**Table:** 

Nomenclature	Androgen receptor	Glucocorticoid receptor
Systematic nomenclature	NR3C4	NR3C1
HGNC, UniProt	*AR* , P10275	*NR3C1* , P04150
Rank order of potency	dihydrotestosterone > testosterone	cortisol, corticosterone≫aldosterone, deoxycortisone [123]
Endogenous agonists	dihydrotestosterone [142]	–
Selective agonists	testosterone propionate [93], mibolerone [50], fluoxymesterone [60], methyltrienolone [148], dromostanolone propionate	fluticasone propionate [10], flunisolide [3], beclometasone [3], methylprednisolone [3], betamethasone [3], budesonide [100]
Selective antagonists	bicalutamide (pK_i_ 7.7) [69], PF0998425 (pIC_50_ 7.1–7.5) [84], enzalutamide (pIC_50_ 7.4) [143], nilutamide (pIC_50_ 7.1–7.1) [131], hydroxyflutamide (pEC_50_ 6.6) [148], galeterone (pIC_50_ 6.4) [56], flutamide (Displacement of ^3^[H] testosterone from wild‐type androgen receptors) (pK_i_ 5.4) [147]	onapristone (pIC_50_ 7.6) [165], ZK112993
Labelled ligands	[^3^H]dihydrotestosterone (Selective Agonist), [^3^H]methyltrienolone (Selective Agonist), [^3^H]mibolerone (Agonist)	[^3^H]dexamethasone (Agonist)

**Table:** 

Nomenclature	Mineralocorticoid receptor	Progesterone receptor
Systematic nomenclature	NR3C2	NR3C3
HGNC, UniProt	*NR3C2* , P08235	*PGR* , P06401
Rank order of potency	corticosterone, cortisol, aldosterone, progesterone [123]	progesterone
Endogenous agonists	deoxycorticosterone [123], aldosterone [57, 123], cortisol [57, 123], corticosterone	progesterone [37]
Selective agonists	–	medroxyprogesterone (Affinity at human PR‐A) [166], ORG2058, levonorgestrel [9, 126]
Selective antagonists	finerenone (pIC_50_ 7.7) [5], eplerenone (pK_i_ 6.9) [6], onapristone (pIC_50_ 6.3) [165], RU28318, ZK112993	ulipristal acetate (pIC_50_ 9.7) [121], mifepristone (Mixed) (pK_i_ 9) [167], onapristone (pK_i_ 7.7) [55], ZK112993
Labelled ligands	[^3^H]aldosterone (Selective Agonist) [44, 135] – Rat	[^3^H]ORG2058 (Selective Agonist)
Comments	Pre‐receptor ligand specificity is provided for the MR in tissues associated with maintenance of sodium homeostasis by the co‐expression of 11β‐hydroxysteroid dehydrogenase type II which converts cortisol (corticosterone in rodents) to their inactive forms. Given the increasing use of *Danio rerio* (zebrafish) as an experimental model, it is important to note that progesterone (and spironolactone) is a MR agonist in fish [42].	–

### Comments

[^3^H]dexamethasone also binds to MR *in vitro*. PR antagonists have been suggested to subdivide into Type I (*e.g.* onapristone) and Type II (*e.g*. ZK112993) groups. These groups appear to promote binding of PR to DNA with different efficacies and evoke distinct conformational changes in the receptor, leading to a transcription‐neutral complex [43, 81]. Mutations in AR underlie testicular feminization and androgen insensitivity syndromes, spinal and bulbar muscular atrophy (Kennedy's disease).

## References

[bph15540-bib-0001] Alexander SPH , *et al.* (2019). [31710718]

[bph15540-bib-0002] Apfel C , *et al.* (1992). [1323127]

[bph15540-bib-0003] Auerbach SS , *et al.* DrugMatrix. Accessed on 02/05/2014.

[bph15540-bib-0004] Baker KD , *et al.* (2003). [12809604]

[bph15540-bib-0005] Bärfacker L , *et al.* (2012). [22791416]

[bph15540-bib-0006] Bell MG , *et al.* (2007). [18038968]

[bph15540-bib-0007] Benoit G , *et al.* (2006). [17132856]

[bph15540-bib-0008] Bergh JJ , *et al.* (2005). [15802494]

[bph15540-bib-0009] Bergink EW , *et al.* (1983). [6645495]

[bph15540-bib-0010] Biggadike K , *et al.* (2000). [10633034]

[bph15540-bib-0011] Bishop JE , *et al.* (1994). [7976510]

[bph15540-bib-0012] Bishop‐Bailey D , *et al.* (2000). [11030710]

[bph15540-bib-0013] Bishop‐Bailey D , *et al.* (2003). [12749590]

[bph15540-bib-0014] Bitsch F , *et al.* (2003). [14622968]

[bph15540-bib-0015] Blumberg B , *et al.* (1998). [9784494]

[bph15540-bib-0016] Boehm MF , *et al.* (1994). [8071941]

[bph15540-bib-0017] Brown KK , *et al.* (1999). [10389847]

[bph15540-bib-0018] Brown PJ , *et al.* (2001). [11354382]

[bph15540-bib-0019] Brun LD , *et al.* (1980). [6777394]

[bph15540-bib-0020] Canan Koch SS , *et al.* (1999). [10052980]

[bph15540-bib-0021] Chambon P (1996). [8801176]

[bph15540-bib-0022] Charpentier B , *et al.* (1995). [8544175]

[bph15540-bib-0023] Charton J , *et al.* (2009). [19058965]

[bph15540-bib-0024] Chiellini G , *et al.* (1998). [9653548]

[bph15540-bib-0025] Collins JL , *et al.* (2002). [11985463]

[bph15540-bib-0026] Colston KW , *et al.* (1992). [1472092]

[bph15540-bib-0027] Cvoro A , *et al.* (2008). [18097065]

[bph15540-bib-0028] Dahlman‐Wright K , *et al.* (2006). [17132854]

[bph15540-bib-0029] De Filippis B , *et al.* (2011). [21889235]

[bph15540-bib-0030] Del Tredici AL , *et al.* (2008). [18055761]

[bph15540-bib-0031] Delescluse C , *et al.* (1991). [1656191]

[bph15540-bib-0032] Doebber TW , *et al.* (2004). [15120604]

[bph15540-bib-0033] Downes M , *et al.* (2003). [12718892]

[bph15540-bib-0034] Eberl G , *et al.* (2004). [15247480]

[bph15540-bib-0035] Eberl G , *et al.* (2004). [14691482]

[bph15540-bib-0036] Ebisawa M , *et al.* (1999). [10748721]

[bph15540-bib-0037] Edwards JP , *et al.* (1998). [9667968]

[bph15540-bib-0038] Erben RG , *et al.* (2002). [12089348]

[bph15540-bib-0039] Flaig R , *et al.* (2005). [15716272]

[bph15540-bib-0040] Flamant F , *et al.* (2006). [17132849]

[bph15540-bib-0041] Fujishima T , *et al.* (2003). [12901907]

[bph15540-bib-0042] Fuller PJ , *et al.* (2019). [31439819]

[bph15540-bib-0043] Gass EK , *et al.* (1998). [9528977]

[bph15540-bib-0044] Ge RS , *et al.* (2005). [16188378]

[bph15540-bib-0045] Géhin M , *et al.* (1999). [10421757]

[bph15540-bib-0046] Germain P , *et al.* (2006). [17132850]

[bph15540-bib-0047] Germain P , *et al.* (2006). [17132853]

[bph15540-bib-0048] Germain P , *et al.* (2009). [19477412]

[bph15540-bib-0049] Germain P , *et al.* (2002). [11805839]

[bph15540-bib-0050] Giwercman A , *et al.* (2000). [10852459]

[bph15540-bib-0051] Graham TL , *et al.* (2005). [15939051]

[bph15540-bib-0052] Grant D , *et al.* (2010). [20677822]

[bph15540-bib-0053] Griffett K , *et al.* (2020). [32763139]

[bph15540-bib-0054] Guo Q , *et al.* (2004). [14701675]

[bph15540-bib-0055] Hamann LG , *et al.* (1996). [8627601]

[bph15540-bib-0056] Handratta VD , *et al.* (2005). [15828836]

[bph15540-bib-0057] Hellal‐Levy C , *et al.* (1999). [10611474]

[bph15540-bib-0058] Henke BR , *et al.* (1998). [9836620]

[bph15540-bib-0059] Herdick M , *et al.* (2000). [11094341]

[bph15540-bib-0060] Higuchi RI , *et al.* (2007). [17439112]

[bph15540-bib-0061] Jain N , *et al.* (2006). [16722623]

[bph15540-bib-0062] Jiang G , *et al.* (1995). [7651430]

[bph15540-bib-0063] Jones SA , *et al.* (2000). [10628745]

[bph15540-bib-0064] Kallen J , *et al.* (2004). [14722075]

[bph15540-bib-0065] Kallen JA , *et al.* (2002). [12467577]

[bph15540-bib-0066] Kane CD , *et al.* (2009). [18971326]

[bph15540-bib-0067] Kanno Y , *et al.* (2013). [23665929]

[bph15540-bib-0068] Keidel S , *et al.* (1994). [8264595]

[bph15540-bib-0069] Kinoyama I , *et al.* (2006). [16420057]

[bph15540-bib-0070] Kiselyuk A , *et al.* (2012). [22840769]

[bph15540-bib-0071] Kojetin D , *et al.* (2011). [21043485]

[bph15540-bib-0072] Kraichely DM , *et al.* (2000). [11014206]

[bph15540-bib-0073] Kuiper GG , *et al.* (1997). [9048584]

[bph15540-bib-0074] Land PW , *et al.* (2003). [12902391]

[bph15540-bib-0075] Le Q , *et al.* (2000). [10723137]

[bph15540-bib-0076] Lee G , *et al.* (2002). [11877444]

[bph15540-bib-0077] Leesnitzer LM , *et al.* (2002). [12022867]

[bph15540-bib-0078] Lehmann JM , *et al.* (1997). [9013544]

[bph15540-bib-0079] Lehmann JM , *et al.* (1997). [9013583]

[bph15540-bib-0080] Lehmann JM , *et al.* (1998). [9727070]

[bph15540-bib-0081] Leonhardt SA , *et al.* (1998). [9849965]

[bph15540-bib-0082] Levin ER (2008). [18784332]

[bph15540-bib-0083] Li E (1999). [10331664]

[bph15540-bib-0084] Li JJ , *et al.* (2008). [18921992]

[bph15540-bib-0085] Lu NZ , *et al.* (2006). [17132855]

[bph15540-bib-0086] Lund BW , *et al.* (2009). [19239230]

[bph15540-bib-0087] Lund BW , *et al.* (2005). [16302793]

[bph15540-bib-0088] Madoux F , *et al.* (2008). [18334597]

[bph15540-bib-0089] Maglich JM , *et al.* (2003). [12611900]

[bph15540-bib-0090] Makishima M , *et al.* (1999). [10334992]

[bph15540-bib-0091] Malamas MS , *et al.* (2004). [15456246]

[bph15540-bib-0092] Maloney PR , *et al.* (2000). [10956205]

[bph15540-bib-0093] Manfredi MC , *et al.* (2007). [17574413]

[bph15540-bib-0094] Mangelsdorf DJ , *et al.* (1995). [8521508]

[bph15540-bib-0095] Matta‐Camacho E , *et al.* (2014). [24872411]

[bph15540-bib-0096] McIntyre TM , *et al.* (2003). [12502787]

[bph15540-bib-0097] Meyers MJ , *et al.* (1999). [10395487]

[bph15540-bib-0098] Meyers MJ , *et al.* (2001). [11708925]

[bph15540-bib-0099] Michalik L , *et al.* (2006). [17132851]

[bph15540-bib-0100] Millan DS , *et al.* (2011). [21880489] 10.4155/fmc.11.96 21942253

[bph15540-bib-0101] Miller CP , *et al.* (2001). [11356100]

[bph15540-bib-0102] Monaghan AP , *et al.* (1997). [9394001]

[bph15540-bib-0103] Moore DD , *et al.* (2006). [17132852]

[bph15540-bib-0104] Moore LB , *et al.* (2000). [10852961]

[bph15540-bib-0105] Moore LB , *et al.* (2000). [10748001]

[bph15540-bib-0106] Nahoum V , *et al.* (2007). [17947383]

[bph15540-bib-0107] Nakamuta M , *et al.* (2002). [11991651]

[bph15540-bib-0108] Nguyen NH , *et al.* (2002). [12109914]

[bph15540-bib-0109] Ning M , *et al.* (2007). [17115070]

[bph15540-bib-0110] Oliver WR , *et al.* (2001). [11309497]

[bph15540-bib-0111] Otte K , *et al.* (2003). [12529392]

[bph15540-bib-0112] Paravicini G , *et al.* (1996). [8858107]

[bph15540-bib-0113] Parks DJ , *et al.* (1999). [10334993]

[bph15540-bib-0114] Pellicciari R , *et al.* (2002). [12166927]

[bph15540-bib-0115] Pereira FA , *et al.* (1999). [10215630]

[bph15540-bib-0116] Prossnitz ER , *et al.* (2008). [18271749]

[bph15540-bib-0117] Prossnitz ER , *et al.* (2009). [19389460]

[bph15540-bib-0118] Qiu Y , *et al.* (1997). [9271116]

[bph15540-bib-0119] Raghuram S , *et al.* (2007). [18037887]

[bph15540-bib-0120] Repa JJ , *et al.* (2000). [10968783]

[bph15540-bib-0121] Rewinkel J , *et al.* (2008). [18243712]

[bph15540-bib-0122] Rocchi S , *et al.* (2001). [11684010]

[bph15540-bib-0123] Rupprecht R , *et al.* (1993). [8282004]

[bph15540-bib-0124] Saito N , *et al.* (2006). [17125259]

[bph15540-bib-0125] Sakamoto J , *et al.* (2000). [11095972]

[bph15540-bib-0126] Schindler AE , *et al.* (2003). [14670641]

[bph15540-bib-0127] Schupp M , *et al.* (2007). [17290005]

[bph15540-bib-0128] Sharma SK , *et al.* (1977). [269396]

[bph15540-bib-0129] Shearer BG , *et al.* (2008). [17975020]

[bph15540-bib-0130] Shyr CR , *et al.* (2002). [12052874]

[bph15540-bib-0131] Simard J , *et al.* (1997). [9111629]

[bph15540-bib-0132] Smith JL , *et al.* (2008). [18603275]

[bph15540-bib-0133] Stauffer SR , *et al.* (2000). [11150164]

[bph15540-bib-0134] Stehlin‐Gaon C , *et al.* (2003). [12958591] 10.1038/nsb979 12958591

[bph15540-bib-0135] Stephenson G , *et al.* (1984). [6320679]

[bph15540-bib-0136] Suetsugi M , *et al.* (2003). [14638870]

[bph15540-bib-0137] Sun J , *et al.* (2002). [11861516]

[bph15540-bib-0138] Sun J , *et al.* (1999). [9927308]

[bph15540-bib-0139] Sun Z , *et al.* (2000). [10875923]

[bph15540-bib-0140] Sznaidman ML , *et al.* (2003). [12699745]

[bph15540-bib-0141] Thacher SM , *et al.* (2000). [10637371]

[bph15540-bib-0142] Tilley WD , *et al.* (1989). [2911578]

[bph15540-bib-0143] Tran C , *et al.* (2009). [19359544]

[bph15540-bib-0144] Tzameli I , *et al.* (2000). [10757780]

[bph15540-bib-0145] Vegeto E , *et al.* (1993). [8264658]

[bph15540-bib-0146] Vizirianakis IS , *et al.* (2010). [20925433]

[bph15540-bib-0147] Wakabayashi K , *et al.* (2008). [18571420]

[bph15540-bib-0148] Wang LG , *et al.* (1998). [10076535]

[bph15540-bib-0149] Wang Y , *et al.* (2000). [11043571]

[bph15540-bib-0150] Wang Z , *et al.* (2003). [12774125]

[bph15540-bib-0151] Warnecke M , *et al.* (2005). [15741322]

[bph15540-bib-0152] Wentworth JM , *et al.* (2000). [10974665]

[bph15540-bib-0153] Wiberg K , *et al.* (1995). [8573413]

[bph15540-bib-0154] Wiesenberg I , *et al.* (1995). [7885826]

[bph15540-bib-0155] Willson TM , *et al.* (2000). [10691680]

[bph15540-bib-0156] Willy PJ , *et al.* (2004). [15184675]

[bph15540-bib-0157] Wu J , *et al.* (2002). [12089353]

[bph15540-bib-0158] Xu HE , *et al.* (2002). [11845213]

[bph15540-bib-0159] Xu Y , *et al.* (2004). [15115385]

[bph15540-bib-0160] Yin L , *et al.* (2007). [18006707]

[bph15540-bib-0161] Young PW , *et al.* (1998). [9454824]

[bph15540-bib-0162] Yu DD , *et al.* (2005). [15713377]

[bph15540-bib-0163] Yuan X , *et al.* (2009). [19440305]

[bph15540-bib-0164] Zhan Y , *et al.* (2008). [18690216]

[bph15540-bib-0165] Zhi L , *et al.* (2003). [12781198]

[bph15540-bib-0166] Zhi L , *et al.* (1998). [9464360]

[bph15540-bib-0167] Zhi L , *et al.* (2003). [12781197]

[bph15540-bib-0168] Zhou HB , *et al.* (2007). [17228884]

[bph15540-bib-0169] Zhou XE , *et al.* (2011). [21068381]

[bph15540-bib-0170] Zhu Y , *et al.* (2003). [12601167]

[bph15540-bib-0171] Zhu Y , *et al.* (2003). [12574519]

[bph15540-bib-0172] Zuercher WJ , *et al.* (2005). [15857113]

